# Sexual Orientation Diversity and Specialty Choice Among Graduating Allopathic Medical Students in the United States

**DOI:** 10.1001/jamanetworkopen.2021.26983

**Published:** 2021-09-30

**Authors:** Westley S. Mori, Yi Gao, Eleni Linos, Mitchell R. Lunn, Juno Obedin-Maliver, Howa Yeung, Matthew D. Mansh

**Affiliations:** 1 Department of Dermatology, University of Minnesota Medical School, Minneapolis; 2 Department of Dermatology, Stanford University School of Medicine, Stanford, California; 3 Division of Nephrology, Department of Medicine, Stanford University School of Medicine, Stanford, California; 4 Department of Epidemiology and Population Health, Stanford University School of Medicine, Stanford, California; 5 Department of Obstetrics and Gynecology, Stanford University School of Medicine, Stanford, California; 6 Department of Dermatology, Emory University School of Medicine, Atlanta, Georgia

## Abstract

This survey study examines the prevalence of gay, lesbian, or bisexual individuals among graduating allopathic medical students by specialty choice in the US.

## Introduction

Sexual minority (SM) people, including bisexual, gay, and lesbian individuals, face numerous health disparities,^1^ including poor access to knowledgeable clinicians and culturally sensitive care.^2,3^ Workforce diversity is essential to ensure a pipeline of physicians equipped through personal experiences and diverse learning environments to improve care for SM people, but little is known about sexual orientation diversity in medical training.^3^ This study aimed to assess the distribution of SM people by intended specialty among graduating medical students.

## Methods

We conducted a survey study of graduating allopathic medical students in the United States using anonymized data pooled from the 2016 to 2019 Association of American Medical Colleges Graduation Questionnaires. This study was deemed exempt by the University of Minnesota as it was a secondary analysis of previously collected, deidentified data. The Association of American Colleges obtained informed consent when originally conducting the questionnaires.

Race and ethnicity data were self-reported. Students were categorized being from racial and ethnic minority groups underrepresented in medicine (URiM) if they reported identifying with at least one of the following: American Indian or Alaska Native; Black or African American; Hispanic, Latino, or of Spanish origin; or Native Hawaiian or other Pacific Islander. Demographics were compared between SM (self-identified as bisexual, gay, or lesbian) and heterosexual (self-identified as heterosexual or straight) students using a Wilcoxon-type trend test (for age only) or χ^2^ tests, and we calculated the percentage of students identifying as SM people by intended specialty. As secondary outcomes in sex-stratified analyses, we compared the percentage of students intending to practice in primary care and surgical specialties, respectively, by SM identity, including interaction analyses (eMethods in the Supplement).

Statistical significance was set at 2-sided *P* < .05. Statistical analysis was performed using STATA statistical software version 16.1 (StataCorp) from September 1 to September 15, 2020.

## Results

Students with missing data on sex or sexual orientation (5051 of 63 721 [7.9%]) or intended specialty (98 of 63 721 [0.2%]) were excluded from the study. Among 58 572 remaining students, 28 724 (49.0%) were female, 55 080 (94.0%) were aged 24 to 32 years, and 8883 (15.2%) identified as being from racial and ethnic minority groups categorized as URiM. Of 58 572 total students, 3664 (6.3% [95% CI, 6.1%-6.5%]) were SM; of 28 724 female students, 1592 (5.7% [95% CI, 5.3%-5.8%) were SM, including 1103 bisexual and 489 gay or lesbian individuals; and of 29 848 male students, 2072 (6.9% [95% CI, 6.7%-7.2%]) were SM, including 432 bisexual and 1640 gay or lesbian individuals. Compared with heterosexual students, SM students were more likely to be male (56.6% [2072 of 3664] vs 50.6% [27 776 of 54 908]; *P* < .001), older (eg, aged <26 years: 41.4% of heterosexual students [22 706 of 54 908] vs 34.9% of SM students [1279 of 3664]; aged >33 years: 5.6% of heterosexual students [3046 of 54 908] vs 8.0% of SM students [263 of 3664]; *P* for trend < .001), and URiM (17.4% [637 of 3664] vs 15.0% [8246 of 54 908]; *P* < .001).

Specialties with the highest and lowest percentage of SM-identified students, respectively, were psychiatry (11.6% [367 of 3165]) and orthopedic surgery (1.8% [45 of 2473]) among all students (Figure A; Table); neurological surgery (8.9% [15 of 169]) and dermatology (1.9% [16 of 836]) among female students (Figure B; Table); and obstetrics and gynecology (18.8% [101 of 538]) and orthopedic surgery (1.0% [21 of 2017]) among male students (Figure C; Table). Compared with their heterosexual peers, SM female students were less likely to intend to practice in primary care specialties (SM female students: 41.2% [653 of 1584] vs heterosexual female students: 46.8% [12 640 of 27 005]; *P* < .001), whereas SM male students were more likely to pursue primary care (SM male students: 38.4% [790 of 29 626] vs heterosexual male students: 34.9% [9630 of 27 005], *P* < .001; *P* for interaction < .001). Compared with their heterosexual peers, SM female students were more likely to intend to practice in surgical specialties (SM female students: 27.2% [430 of 1584] vs heterosexual female students: 24.7% [6676 of 27 005]; *P* < .001), whereas SM male students were less likely to pursue surgical specialties (SM male students:17.8% [366 of 2056] vs heterosexual male students: 26.5% [7303 of 27 750]; *P* < .001; *P* for interaction < .001).

**Figure.**
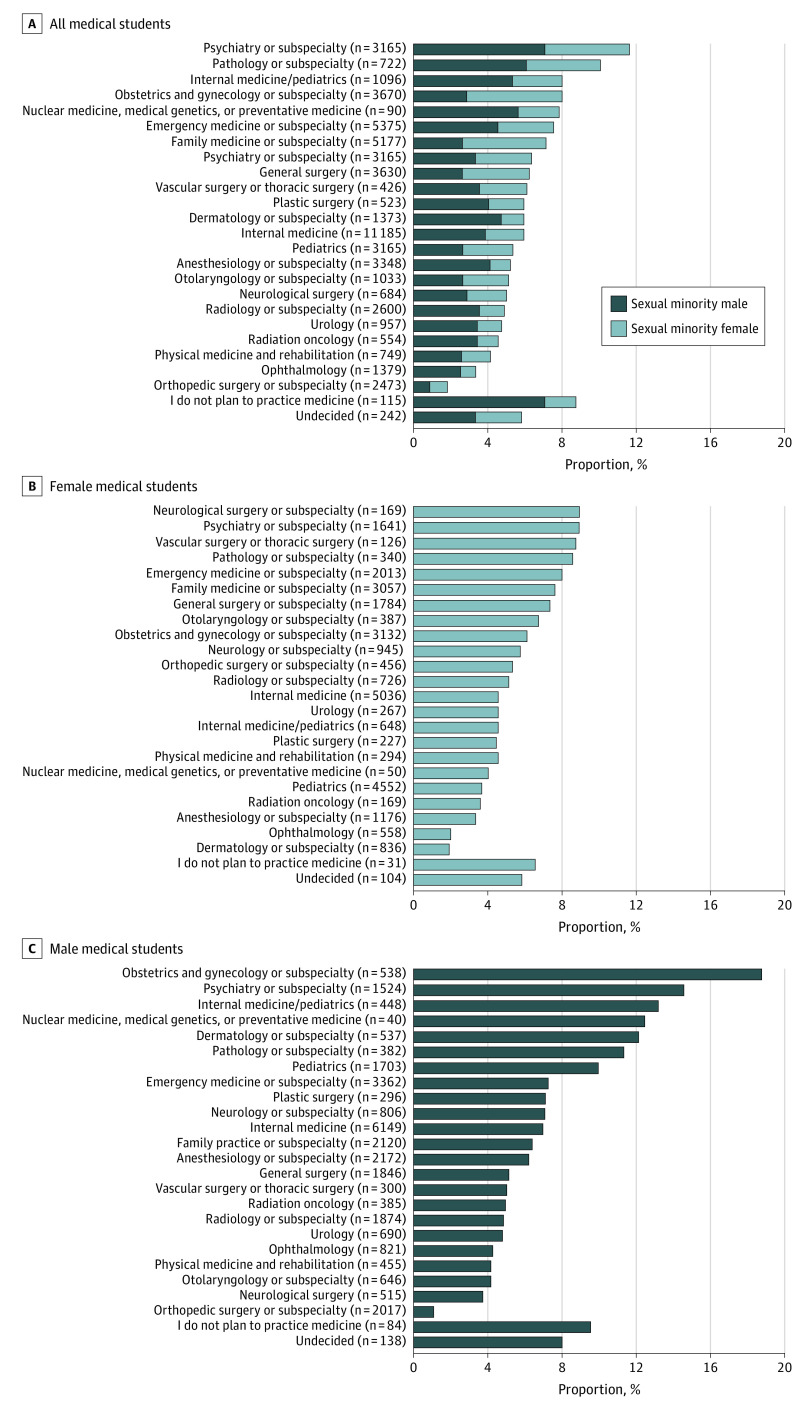
Percentage of Medical Students Identifying as a Sexual Minority by Intended Specialty The figure shows the percentage of medical students identifying as a sexual minority by intended specialty among all medical students (A), female medical students (B), and male medical students (C).

**Table.** Percentage Identifying as a Sexual Minority by Intended Specialty Among All, Female, and Male Graduating Allopathic Medical Students in the US

Intended specialty	Students, No./total No. (%)		
	All medical students (n=58 572)	Female medical students (n=28 724)	Male medical students (n=29 848)
Primary care specialties	1443/23 713 (6.1)	653/13 293 (4.9)	790/10 420 (7.6)
Family practice or subspecialty	366/5177 (7.1)	231/3057 (7.6)	135/2120 (6.4)
Internal medicine	656/11 185 (5.9)	227/5036 (4.5)	429/6149 (7.0)
Internal medicine/pediatrics	88/1096 (8.0)	29/648 (4.5)	59/448 (13.2)
Pediatrics	333/6255 (5.3)	166/4552 (3.7)	167/1703 (9.8)
Surgical specialties	796/14 775 (5.4)	430/7106 (6.1)	366/7669 (4.8)
General surgery	224/3630 (6.2)	130/1784 (7.3)	94/1846 (5.1)
Neurological surgery or subspecialty	34/684 (5.0)	15/169 (8.9)	19/515 (3.7)
Obstetrics and gynecology or subspecialty	292/3670 (8.0)	191/3132 (6.1)	101/538 (18.8)
Ophthalmology	46/1379 (3.3)	11/558 (2.0)	35/821 (4.3)
Orthopaedic surgery or subspecialty	45/2473 (1.8)	24/456 (5.3)	21/2017 (1.0)
Otolaryngology or subspecialty	53/1033 (5.1)	26/387 (6.7)	27/646 (4.2)
Plastic surgery	31/523 (5.9)	10/227 (4.4)	21/296 (7.1)
Vascular surgery or thoracic surgery	26/426 (6.1)	11/126 (8.7)	15/300 (5.0)
Urology	45/957 (4.7)	12/267 (4.5)	33/690 (4.8)
All other specialties	1401/19 727 (7.1)	501/8190 (6.1)	900/11 537 (7.8)
Anesthesiology or subspecialty	175/3348 (5.2)	39/1176 (3.3)	136/2172 (6.3)
Dermatology or subspecialty	81/1373 (5.9)	16/836 (1.9)	65/537 (12.1)
Emergency medicine or subspecialty	404/5375 (7.5)	161/2013 (8.0)	243/3362 (7.2)
Neurology or subspecialty	111/1751 (6.3)	54/945 (5.7)	57/806 (7.1)
Nuclear medicine, medical genetics or preventative medicine	7/90 (7.8)	2/50 (4.0)	5/40 (12.5)
Pathology or subspecialty	72/722 (10.0)	29/340 (8.5)	43/382 (11.3)
Physical medicine and rehabilitation	31/749 (4.1)	12/294 (4.1)	19/455 (4.2)
Psychiatry or subspecialty	367/3165 (11.6)	145/1641 (8.8)	222/1524 (14.6)
Radiology or subspecialty	128/2600 (4.9)	37/726 (5.1)	91/1874 (4.9)
Radiation oncology	25/554 (4.5)	6/169 (3.6)	19/385 (4.9)
I do not plan to practice medicine	10/115 (8.7)	2/31 (6.5)	8/84 (9.5)
Undecided	14/242 (5.8)	6/104 (5.8)	8/138 (5.8)

## Discussion

To our knowledge, this is the first study to examine sexual orientation diversity and specialty choice using a representative sample of medical students. Compared with the general population, wherein 9.4% of female individuals and 5.3% of male individuals aged 25 to 34 years are estimated to identify as lesbian, gay, bisexual, or transgender,^4^ our findings suggest that SM female students may be underrepresented in undergraduate medical training.

SM diversity varied significantly by intended specialty, and we found sex differences across primary care and surgical specialties. SM medical students experience higher levels of mistreatment^5^ and are less likely to pursue specialties perceived to be noninclusive,^6^ indicating specialty-specific training climates could contribute to these differences and that these may vary by sex.

This study had some limitations. These include the lack of data on gender identity, delineation of gender from sex assigned at birth, and analyses addressing intersectionality between multiple minority identities, all of which deserve further attention.

SM people, particularly SM female students, may be underrepresented among medical students overall and in certain specialties. Further research is needed to better understand factors influencing these disparities. Sexual orientation data collection should be standardized in all physician workforce surveys and SM diversity considered in undergraduate and graduate medical training recruitment to promote a diverse physician workforce across all specialties.

## Notes

Supplement. **eAppendix.** Supplemental Methods
eReferences
