# Supplementary material for: Sexual Orientation Diversity and Specialty Choice Among Graduating Allopathic Medical Students in the United States
Source: JAMA Netw Open. 2021 Sep 30;4(9):e2126983. doi: 10.1001/jamanetworkopen.2021.26983 (PMC8485175; doi:10.1001/jamanetworkopen.2021.26983)
Supplement: Supplement. — eAppendix. Supplemental Methods eReferences [file jamanetwopen-e2126983-s001.pdf]

Supplemental Online Content

Mori WS, Gao Y, Linos E, et al. Sexual orientation diversity and specialty choice among graduating allopathic medical students in the United States. *JAMA Netw Open.* 2021;4(9):e2126983. doi:10.1001/jamanetworkopen.2021.26983

eAppendix. Supplemental Methods

eReferences

This supplemental material has been provided by the authors to give readers additional information about their work.

© 2021 Mori WS et al. *JAMA Network Open.*

eAppendix. Supplemental Methods

This study used data from the 2016, 2017, 2018, and 2019 American Association Medical College (AAMC) Graduation Questionnaires (GQ), which are distributed annually to all graduating medical students attending an accredited allopathic medical school in the United States. The GQ surveys had response rates of 80.5%, 81.1%, 83.0%, and 83.6%, in 2016, 2017, 2018, and 2019,[1-4] respectively, which follow American Association for Public Opinion Research (AAPOR) guidelines for calculating and reporting response rates.

Sex, age, and race were populated from previously collected AAMC data sources such as the American Medical College Application Service (AMCAS). The study did not have access to data on the sex assigned at birth or gender identity of students. Sex was classified based on the following survey prompt: "Sex" with "Male" or "Female" being the only two options provided. Age was provided as a categorical variable to ensure the dataset was unidentifiable. Racial/ethnic minorities underrepresented in medicine (URiM) were defined as students identifying as “American Indian or Alaska Native,” “Black or African American,” “Hispanic, Latino, or of Spanish origin,” or “Native Hawaiian or Other Pacific Islander.” Students who chose multiple race/ethnicities were classified as URiM if one of their responses fell within these categories.

Sexual minority (SM) students were defined as those students selecting “bisexual” or “gay or lesbian,” and heterosexual students were defined as those students selecting “heterosexual or straight” in response to the question “How do you self-identify?” These were the only sexual orientation categories provided and respondents were asked to select a single category.

Intended specialty choice was based on the student’s response to the question “When thinking about your career, what is your intended area of practice?” Due to a smaller number of respondents, (1) “vascular surgery” and “thoracic surgery,” and (2) “nuclear medicine,” “medical genetics,” and “preventative medicine” were combined into single groups, respectively. Primary-care specialties were defined as “family practice or subspecialty,” “internal medicine or subspecialty,” “pediatrics or subspecialty,” and “internal medicine/pediatrics.” We did not discriminate between students’ intents on planning on pursuing a subspecialty in one of those primary-care fields. Surgical specialties were defined as “neurological surgery,” “obstetrics and gynecology,” “ophthalmology,” “orthopaedic surgery or subspecialty,” “otolaryngology or subspecialty,” “plastic surgery,” “general surgery,” “vascular surgery,” “thoracic surgery,” and “urology.” Intent to pursue careers in primary-care and surgical specialties were specifically evaluated as secondary outcomes as these are important measures for evaluating overall trends in the career goals of medical students[5] and prior studies have demonstrated that perceived inclusiveness of sexual minorities varies between primary-care and surgical specialties.[6-8]

© 2021 Mori WS et al. *JAMA Network Open.*

For statistical analyses, we first calculated the percentage of graduating medical students who identified as sexual minorities among all, female, and male graduating medical students, including 95% exact (or Clopper-Pearson) binominal confidence intervals. For bivariate analyses comparing demographics by sexual orientation, Wilcoxon-type trend tests (age only) or Pearson's χ2-tests (all other variables) were used to calculate p-values. For analyses assessing the percentage of students who selected primary-care or surgical specialties, respectively, students who selected "I do not plan to practice medicine" (115/58,572; 0.2%) or who were undecided on their intended specialty (242/58,572; 0.4%) were excluded and Pearson's χ2-tests were used to calculate p-values. Interaction analyses were conducted using logistic regression analyses using bivariate outcomes (yes vs. no for choosing either a primary-care or surgical specialty, respectively) controlling for sex (male, female), sexual orientation (heterosexual, sexual minority), and the two-way interaction term between sex and sexual orientation. We report p-values for the two-way interaction term in the logistic regression model. All analyses were conducted using STATA version 16.1 (StataCorp, College Station, TX) with 2-sided α=0.05.

eReferences

1. Association of American Medical Colleges. Medical School Graduation Questionnaire: 2016 All Schools Summary Report. https://www.aamc.org/system/files/reports/1/2016gqallschoolssummaryreport.pdf. Published July 2016. Accessed October 27, 2020.
2. Association of American Medical Colleges. Medical School Graduation Questionnaire: 2017 All Schools Summary Report. https://www.aamc.org/ system/files/reports/1/ 2017gqallschoolssummaryreport.pdf. Published July 2017. Accessed October 27, 2020.
3. Association of American Medical Colleges. Medical School Graduation Questionnaire: 2018 All Schools Summary Report. https://www.aamc.org/ system/files/reports/1/ 2018gqallschoolssummaryreport.pdf. Published July 2018. Accessed October 27, 2020.
4. Association of American Medical Colleges. Medical School Graduation Questionnaire: 2019 All Schools Summary Report. https://www.aamc.org/system/files/2019-08/2019-gq-all-schools-summary-report.pdf. Published July 2019. Accessed October 27, 2020.
5. Newton DA, Grayson MS. Trends in career choice by US medical school graduates. JAMA. 2003;290(9):1179-1182.
6. Sitkin NA, Pachankis JE. The Role of Specialty Prestige, Perceived Inclusion, and Medical School Climate. *LGBT Heal*. 2016;3(6):451-460.
7. Mansh M, White W, Gee-Tong L, et al. Sexual and gender minority identity disclosure during undergraduate medical education: "in the closet" in medical school. Acad Med. 2015;90(5):634-644.
8. Lee KP, Kelz RR, Dubé B, Morris JB. Attitude and perceptions of the other underrepresented minority in surgery. J Surg Educ. 2014;71(6):e47-52.

© 2021 Mori WS et al. *JAMA Network Open.*

© 2021 Mori WS et al. *JAMA Network Open.*
